# Synthesis and Self-Assembly of Shape Amphiphiles Based on POSS-Dendron Conjugates

**DOI:** 10.3390/molecules22040622

**Published:** 2017-04-21

**Authors:** Yu Shao, Minyuan Ding, Yujie Xu, Fangjia Zhao, Hui Dai, Xia-Ran Miao, Shuguang Yang, Hui Li

**Affiliations:** 1 State Key Laboratory for Modification of Chemical Fibers and Polymer Materials and College of Material Science and Engineering, Center for Advanced Low-Dimension Materials, Donghua University, Shanghai 201620, China; boukephalas@outlook.com; 2 School of Materials Science and Engineering, East China University of Science and Technology, Shanghai 200237, China; haristerteam@163.com (M.D.); xuyujiejack@163.com (Y.X.); mailhappy@foxmail.com (F.Z.); stable_fyi@163.com (H.D.); 3 Shanghai Institute of Applied Physics, Chinese Academy of Science, Shanghai 201204, China; miaoxiaran@sinap.ac.cn

**Keywords:** shape amphiphiles, dendrimer, polyhedral oligomeric silsesquioxane, hierarchical structure

## Abstract

Shape has been increasingly recognized as an important factor for self-assembly. In this paper, a series of shape amphiphiles have been built by linking polyhedral oligomeric silsesquioxane (POSS) and a dendron via linkers of different lengths. Three conjugates of octahedral silsesquioxanes (T_8_-POSS) and dendron are designed and synthesized and are referred to as isobutyl T_8_-POSS gallic acid derivatives (BPOSS-GAD-1, BPOSS-GAD-2, BPOSS-GAD-3). These samples have been fully characterized by ^1^H-NMR, ^13^C-NMR, Fourier transform infrared (FT-IR) spectroscopy and matrix-assisted laser desorption/ionization time of flight (MALDI-TOF) mass spectrometry to establish their chemical identity and purity. Driven by different interactions between POSS and dendron, ordered superstructure can be found upon self-assembly. The stabilities and structures of these samples are further studied by using differential scanning calorimetry (DSC), small-angle X-ray scattering (SAXS), wide-angle X-ray diffraction (WAXD), and molecular simulations. The results show that their melting points range from 74 °C to 143 °C and the molecular packing schemes in the assemblies can form lamellar structure of BPOSS-GAD-3 as determined by the different linkers.

## 1. Introduction

Shape amphiphiles are conjugates of at least two molecular segments of distinct shapes, such as spheres, rods, triangles, discs, etc., which may or may not bear different interaction features such as hydrophobic, hydrophilic, or omniphobic groups [1,2]. Anisotropy of shape amphiphiles is the major driving force for their self-assembly into well-defined structures [3]. Traditional shape amphiphiles include random-coil block copolymers (if no shape is also considered as a special type of shape) [4], protein-polymer hybrids (where folded proteins usually possess a well-defined overall shape) [5], or conjugates of molecular particles possessing consistent shape like rods, spheres, and disks [6]. With the burgeoning research interest in these class of molecules, a roadmap for their self-assembly have been given by Damasceno et al. via a systematic work of simulation on the self-assembly of different polyhedral [7] and various tethered nano-building blocks demonstrated by Zhang et al. [8]. Among all different kinds of shape-persistent molecules, dendrimers and molecular nanoparticles show uniquely intriguing properties. In the past two decades, dendrimers have attracted lots of attentions because of their predictable and tunable properties. They can assemble into complex macromolecular superstructures. Nevertheless, synthesis of dendrimers usually requires demanding experiment techniques and delicate design to yield high-generation defect-free, monodisperse samples with relatively large molecular weights. Gallic acid can be viewed as a simple type of low-generation dendron. Unique physical properties had been demonstrated by Hudson et al. on dendrimers bearing gallic acid building blocks [9]. It was found that the first generation dendron was crystalline, and higher-generation dendrons could show different kinds of liquid-crystalline (LC) phases ranging from cylindrical to spherical phases. Later, it was further revealed that these kinds of dendrimers could assemble into nontraditional super-lattices such as the Frank-Kasper A_15_ phase [10,11]. On the other hand, molecular nanoparticles, described by Zhang et al. as shape- and volume-persistent nano-objects with well-defined molecular structure and specific symmetry [12], are shown to be highly versatile building blocks for shape amphiphiles.

Molecular nanoparticles typically include folded globular proteins, polyhedral oligomeric silsesquioxane (POSS), fullerene (C_60_), and polyoxometalates (POMs). Because of their incompressible and impenetrable features, they can act as “nano-atoms” to build giant molecules that are prototype precision synthetic macromolecules that are monodisperse. Among all these kinds of molecular nanoparticles, POSS has drawn special research interest. POSS is arguably the smallest silica nanoparticles that possesses a well-defined, covalent cage structure as the molecular scaffold and their diameters are −1 nm. Up to date, a large number of substituents have been successfully appended to the vertexes of the POSS cages [R(SiO_1.5_)]*_n_* (*n* = 8, 10, 12, and larger), but most of them are inert, such as isopropyl, isooctyl, cyclopentyl, phenyl, vinyl, perfluorinated alkyl, and chloropropyl groups [13]. It is noteworthy that many of these compounds are commercially available. Owning to the facile synthesis and unique O_h_ symmetry of T_8_-POSS cage derivatives, they are versatile building blocks that have been frequently used and studied by materials scientists. In particular, many shape amphiphiles had been synthesized bearing T_8_-POSS as the cubic motif and their phase behaviors have been thoroughly studied in solution, in bulk and in thin film. They include sphere-cube like C_60_-POSS conjugates [14], sphere-rod like C_60_-oligfluorene conjugates [15], sphere-disk like C_60_-porphyrin conjugates [16,17,18], sphere-plane like C_60_-perylene diimide (PDI) conjugates, [19] sphere-pyramid like C_60_-triphenylamine conjugates [20], cube-plate like POSS-HBC conjugates [21], cube-plane-cube like POSS-PDI-POSS conjugates [22], just to name a few. Particularly, in bulk phase, hierarchical structures with sub-10 nm feature could be easily obtained in these giant molecules [23]. More strikingly, Cheng et al. demonstrated that giant tetrahedra constructed using different POSS cages linked to a tetrahedral core can generate thermodynamically stable supramolecular A_15_ lattices, which remains uncommon for such a rigid molecule [24]. Cui et al. also reported the supramolecular self-assembly liquid crystalline and crystalline structures of disc-cubic shape amphiphiles bearing triphenylene and POSS [25]. In solution, shape amphiphiles can also achieve unique structures especially the 1D nanobelts and 2D nanosheets. Liu et al. demonstrated that sphere-sphere shape amphiphiles of POM-POSS, where POM are highly charged, tend to form 2D nanocrystals with an exact thickness of a double layer [26]. More recently, they further reported that the dimension of α-Keggin-type polyoxometalate POSS conjugate (KPOM-POSS) self-assemblies can be tuned by the environment to form 1D nanobelts, 2D nanosheets, multiple-layered stacked crystals and inner curved lamellae [27]. In order to gain insight into the effect of linker type and length on the self-assembly of shape amphiphiles, we designed and synthesized a series of POSS-dendron conjugates. In this contribution, we report our study on the synthesis and characterization of a series of such shape amphiphiles based on the conjugation of POSS and dendron in the effort to understand and tune the properties of these materials via design.

## 2. Results

### 2.1. Molecular Synthesis and Characterizations

**Scheme 1:**
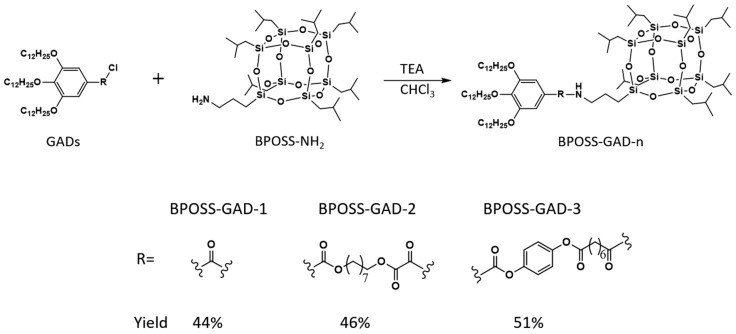
Synthesis route of the target shape amphiphiles.

Scheme 1 shows the synthetic route to three kinds of shape amphiphiles bearing cubic T_8_-POSS and branched dendron. All these derivatives were synthesized through well-established methods such as esterification, etherification, acylation and amidation reactions [28]. First of all, gallic acid was reacted with excess amount of methanol under concentrated H_2_SO_4_ catalysis to give gallic ester in 66% yield. Then all three phenolic hydroxyl groups were reacted with bromododecane under nitrogen to give the gallic ester dendron in 62% yield. Following the hydrolysis of the esters to give the free carboxylic acids, three carboxyl chlorides were prepared to react with the amine functional group on isobutyl-functionalized POSS (BPOSS) to give the final shape amphiphiles in decent yields ranging from 44%–51%.

**Figure 1:**
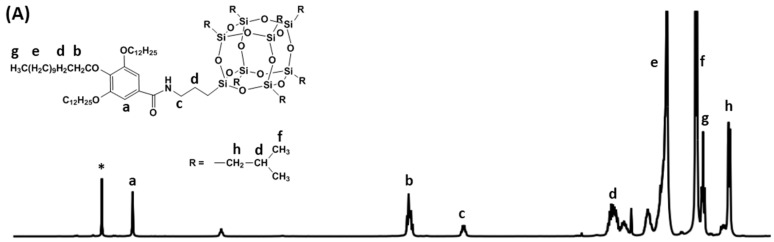

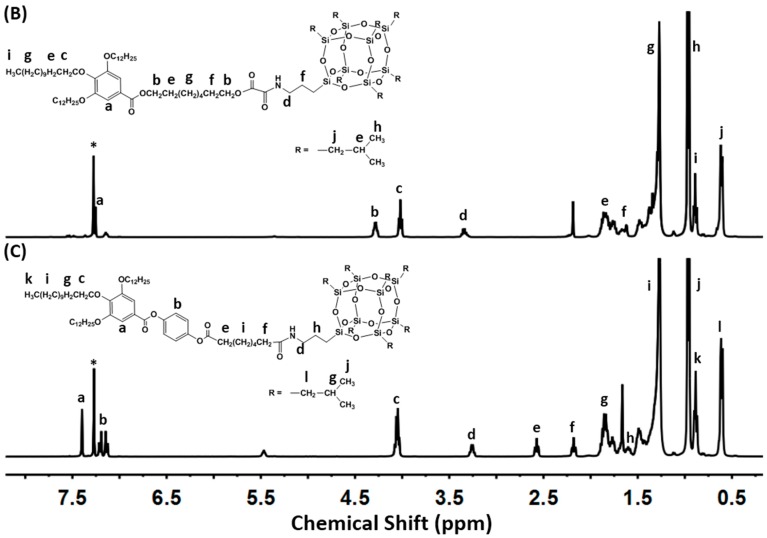
^1^H-NMR spectra of BPOSS-GAD-1 (**A**), BPOSS-GAD-2 (**B**) and BPOSS-GAD-3 (**C**). Asterisk (*) represents the resonances from the residual proton signals from CDCl_3_.

Figure 1 shows the ^1^H-NMR results of three isobutyl T_8_-POSS gallic acid derivatives (BPOSS-GAD-1, BPOSS-GAD-2, BPOSS-GAD-3). Proton resonances could be unambiguously assigned. Clearly, there are three proton regions in each spectrum: the first region refers to the aromatic protons at about 7.0 ppm; the second ranges from 2.0 to 4.5 ppm, which can be attributed to protons adjacent to carboxyl groups, amino groups, hydroxyl groups or ester groups; the third is the proton resonances of alkyl groups at 2.0 ppm or less. The ratios of the integrated areas of each type of protons also matches the expected values (for details, see Supporting Information). The corresponding ^13^C-NMR spectra (Figure S1) exhibit characteristic peaks as expected. The successful conjugation of POSS and gallic acid were also confirmed by FT-IR spectrometry (Figures S2–S4), where the strongest peak at 1110 cm^−1^ is attributed to the stretching vibration of Si–O–Si in the POSS core and the peaks of carboxyl groups are at 1640 cm^−1^ and 1750 cm^−1^. In order to further prove the chemical structures of the molecules, MALDI-TOF mass spectra were obtained under reflection mode for each compound as shown in Figures S5–S7. For BPOSS-GAD-1, the calculated molecular weight for monoisotropic peak of NaC_74_H_147_NO_16_Si_8_ is 1552.88 Da, and the found experiment *m*/*z* value was 1552.97. The other two conjugates, also have the right *m*/*z* values of 1753.64 and 1800.88 for BPOSS-GAD-2 and BPOSS-GAD-3, respectively, as anticipated.

### 2.2. Structures in the Condensed State

Dendrons bearing gallic acid and other higher-generation dendrimers are potent liquid crystalline mesogens due to their rigid shape with appropriate aspect ratios. By contrast, BPOSS always tends to crystallize because of its high symmetry. It is thus very intriguing to see which parts would win the competition and dominate the structure formation. Therefore, their phase structures were carefully studied using a combination of different techniques, including differential scanning calorimetry (DSC), polarized light microscopy (PLM), small-angle X-ray scattering (SAXS) and wide-angle X-ray diffraction (WAXD), and the molecular packing models were finally simulated using Material Studio.

**Figure 2:**
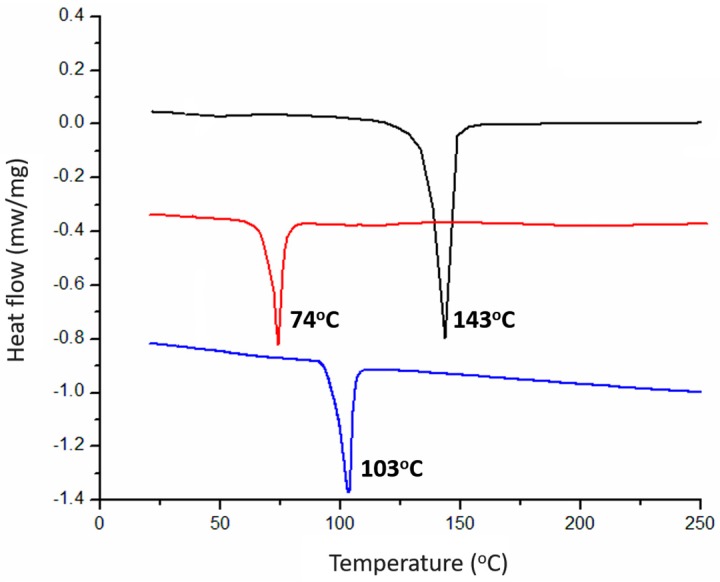
DSC curves of BPOSS-GAD-1 (black line), BPOSS-GAD-2 (red line) and BPOSS-GAD-3 (blue line).

The phase behavior of these shape amphiphiles was first examined by DSC at a heating/cooling rate of 10 °C/min. The resulting thermograms are shown in Figure 2. On each DSC curve, a first-order transition can be clearly seen, which indicates the melting point of each shape amphiphile. The attachment of the dendron with long, soft alkyl chains which have a low melting point about 39.5–45.5 °C [29] dramatically decreases the melting points of these compounds as compared to BPOSS-NH_2_ (245 °C [30]). This is mainly attributed to two factors: (1) the introduction of dendron breaks the symmetry of POSS and also decreases the rigidity of the molecules and (2) the presence of soft linkers that decrease the segregation of POSS and lower the tendency of POSS toward crystallization. It is evident that BPOSS-GAD-2 bearing the most flexible linker has the lowest melting point of 74 °C. Comparing BPOSS-GAD-2 and BPOSS-GAD-3, we can see that the addition of one benzene ring as part of the linker increases the melting point from 74 °C to 103 °C. In this case, rigidity of benzene ring and the resulting π-π stacking leads to sharp increase in melting point. By further shortening the length of the spacer in BPOSS-GAD-1, the melting point increases again to 143 °C. In this way, the melting points of these shape amphiphiles are facilely tuned.

The evidence of crystal-melt transition also comes from polarizing light microscopy under different temperatures (see Supporting Information Figures S8–S10). By heating above the melting points of these samples, the images appear dark which suggests a homogeneous melt. After cooling to 30 °C, they showed up as spherulites with different sizes. BPOSS-GAD-1 and BPOSS-GAD-3 gives big spherulites whereas BPOSS-GAD-2 only forms very small crystallites. These results collaborate with the DSC data that no liquid crystalline phases exist for all of these samples and BPOSS-GAD-1 and 3 have a higher tendency toward crystallization than BPOSS-GAD-2 due to shorter and more rigid linkers.

**Figure 3:**
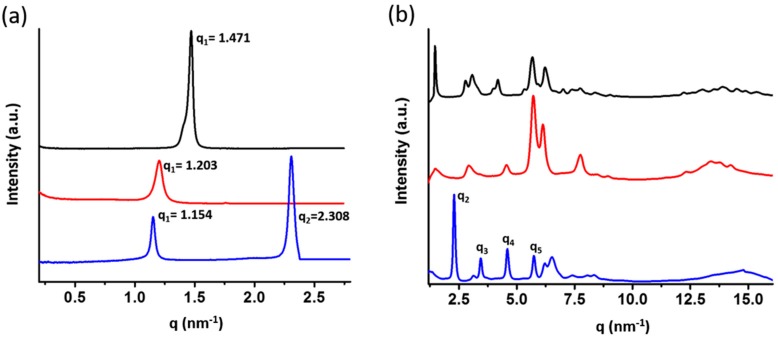
SAXS (**a**) and WAXD (**b**) curves of BPOSS-GAD-1 (lack line), BPOSS-GAD-2 (red line) and BPOSS-GAD-3 (blue line).

From these results, we know that POSS plays a dominating role in determining the crystal structure of these shape amphiphiles in the condensed state. In order to further illustrate the phase structure of these shape amphiphiles, in situ X-ray scattering experiments were carried out for each sample. Before the X-ray experiments, the samples were heated up above their melting points to completely eliminate the thermal history and then cooled and annealed at a temperature ten degrees lower than their corresponding melting points for 24 h to fully develop the crystal structure. As shown in Figure 3a, sharp diffraction peaks can be observed in the SAXS profiles, which indicates that there is long-range order of molecular packing for each of these compounds in solid state. The structure of BPOSS-GAD-3 can be clearly assigned as lamellar since the values of their diffraction peaks are *q*_1_ = 1.154 (nm^−1^) and *q*_2_ = 2.308 (nm^−1^) with a ratio of 1:2 (Figure 3a). The higher order peaks of *q*_3_, *q*_4_, and *q*_5_ can be further found in the WAXD profiles (Figure 3b) that are integral times of *q*_1_.

For the other two compounds, layer structure is not that clear. The sharp peaks in the WAXD patterns of BPOSS-GAD-1 and BPOSS-GAD-2 suggest that they are highly ordered crystals. The peaks around 6 nm^−1^ are crystalline structure of POSS cages which agrees with their size of −1 nm. Higher peaks around 12 nm^−1^ represent the packing of the alkyl chains. The *d*-spacing of these compounds calculated from *q*_1_ are their feature sizes (Table 1).

**Figure 4:**
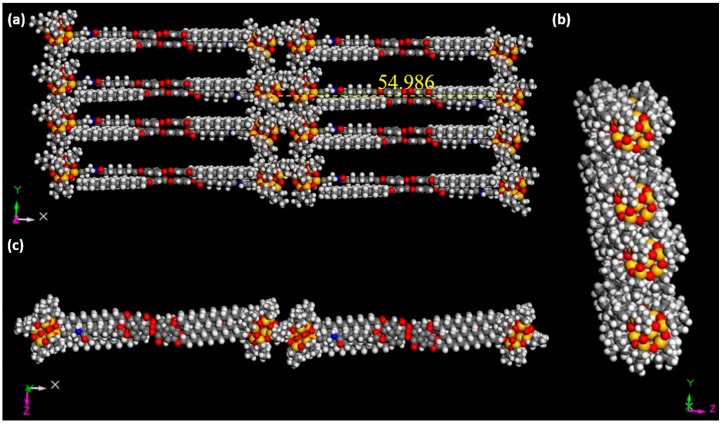
Possible model for the molecular packing scheme of BPOSS-GAD-3 in the lamellae superlattice: (**a**) *xy*-plane when projected from *z*-axis; (**b**) *yz*-plane when projected from *x*-axis; (**c**) *xz*-plane when projected from *y*-axis.

It is clear that with the increasing chain length of the linker, the *d*-spacing also increases from BPOSS-GAD-1 to BPOSS-GAD-3. However, it is not possible to pin down their crystal structures from 1D SAXS and WAXD profiles only. Attempts to grow single crystals of decent sizes to solve their structures and elucidate the molecular packing in the crystals fail to date, probably due to the presence of multiple alkyl chains. To gain insight into their molecular packing, we used computer simulation to study the possible molecular packing scheme of BPOSS-GAD-3 in the condensed state, which seems to be the simplest case among the three. The long axis of the molecules is −2.7 nm, which is roughly half of the long period. We speculated that the lamellae superlattice may be formed by a head-to-head, tail-to-tail packing of the shape amphiphile. Similar packing schemes have been observed in many POSS-containing shape amphiphiles, such as POSS-C_60_, BPOSS-APOSS, POSS-POM, etc. [14,26,27,31]. The crystallization of BPOSS is believed to be the strong driving force for the assembly, which is also responsible for the flat interface. We thus assumed that the BPOSS-GAD-3 takes this arrangement to form the lamellar superlattice and built such a packing in Materials Studio using the following steps: first, we draw this molecule with the software and use “Discover mode” to minimize the energy. Then we run geometry optimization under the “Forcite mode” several times until the convergence is stable. All energy calculation methods were based on the “Compass” option. At last, we propose the most possible molecular packing model using this structure. The results are shown in Figure 4 where the projections of *xy* plane, *yz* plane, and *xz* plane are shown in (a), (b), and (c), respectively. The lamellae structure along the *x*-axis direction is apparent. Besides the crystallization of BPOSS, the conformational asymmetry is also one of the reason the dendron and POSS pack in different regions. In this case, the simulation gives a *d*-spacing of −54.98 Å which is in good agreement with the tested *d*-spacing (54.42 Å) from SAXS experiment. However, we could not gain more information on the packing of the molecules within the layer. Since the three shape amphiphiles have very similar structures, we anticipated that their molecular packing should also be similar. As for BPOSS-GAD-1, the short and rigid linker may confine BPOSS and the dendron whereas the two components may be decoupled in BPOSS-GAD-3. The strong correlation between BPOSS and GAD may lead to higher order crystal structures. As for BPOSS-GAD-2, the linker is very flexible and long, the structure may be difficult to develop, as seen in the broad X-ray diffraction peaks and the small-sized spherulites under optical microscope. The introduction of phenyl group in the linker is believed to be critical which improves the interaction between the dendron parts and stabilize the structure toward superlattice formation.

## 3. Materials and Methods

### 3.1. Chemicals and Instrumentation

All reagents (analytical grade and spectroscopic grade) were obtained commercially and used as received unless otherwise mentioned, and solvents like Dimethylformamide (DMF), CH_2_Cl_2_, CHCl_3_ were reagent-grade and were dried and freshly distilled before use. NMR spectra were recorded on an ARX400 spectrometer (Bruker, Billerica, MA, USA) at 400 MHz for ^1^H-NMR and 100 MHz for ^13^C-NMR. Chemical shifts (δ values) were reported in ppm, residual resonances from CDCl_3_ was set as 7.27 ppm for ^1^H-NMR and 77.00 ppm for ^13^C-NMR. FT-IR spectra were recorded on Magna-IR instrument (Nicolet, Madison, WI, USA). MALDI-TOF-MS data were recorded on an Axima CFR Plus instrument (Kratos, Manchester, UK) using 1,8,9-triacetoxyanthracene and 2,5-dihydroxybenzoic acid (DBA) as matrix. DSC curves were recorded on a PC200 DSC instrument (NETZSCH, Selb, German) calibrated using indium before use. Heating rate is 10 °C/min and test range is from −90 °C to 400 °C. Polarized light microscopy was done using XPL-30TF microscope (Shanghai WeiTu Optics & Electron Technology Co., Ltd., Shanghai, China) equipped with a hot stage and recorded by a NIKON 100 camera (NIKON, Tokyo, Japan). SAXS/WAXD data was recorded at Beamline 16B at the Shanghai Synchrotron Radiation Facility with the incident wave length of 1.24 nm and a sample-to-detector distance of 2.0 m for SAXS and 24.6 cm for WAXD. Computer simulation was performed on Materials Studio (Accelrys, San Diego, CA, USA).

### 3.2. General Synthetic Procedure

First, carboxylic acids were dissolved in anhydrous CH_2_Cl_2_ and one drop of DMF was added as catalyst. Then, oxalyl chloride was added dropwise in 30 min. When the reaction is completed, the oxalyl chloride and solvents were removed under reduced pressure. The acyl chlorides were used without further purification. The corresponding acyl chloride was dissolved in anhydrous CHCl_3_. Triethylamine was added as the base. Then, a solution of POSS-NH_2_ in anhydrous CHCl_3_ was added dropwise in 30 min. The reaction was stirred at room temperature and monitored by thin layer chromatography (TLC). After completion, the samples were worked-up and further purified through silica gel chromatography.

*3,4,5-tris(Dodecyloxy)-N-(3-(3,5,7,9,11,13,15-heptaisobutyl-2,4,6,8,10,12,14,16,17,18,19,20-dodecaoxa-1,3,5,7,9,11,13,15-octasilapentacyclo[9.5.1.1^3,9^.1^5,15^.1^7,13^]icosan-1-yl)propyl)benzamide (BPOSS-GAD-1)*. Pale yellow powder. Yield: 45%. ^1^H-NMR (CDCl_3_, ppm): δ 6.94 (s, 2H), 4.03–3.97 (m, 6H), 3.44 (dt, *J*_1_ = 6.6 Hz, *J*_2_ = 6.6 Hz, 2H), 1.89–1.69 (m, 17H), 1.27 (br, 56H), 0.97 (d, *J* = 6.6 Hz, 42H), 0.89 (t, *J* = 6.4 Hz, 9H), 0.62 (dd, *J*_1_ = 2.5 Hz, *J*_2_ = 2.5 Hz, 14H). ^13^C-NMR (CDCl_3_, ppm): δ 167.34, 153.05, 141.01, 129.90, 105.63, 73.48, 69.37, 42.40, 31.91, 31.41, 30.92, 30.29, 30.15, 29.73, 29.69, 29.65, 29.63, 29.58, 29.39, 29.36, 26.07, 25.67, 23.87, 23.83, 23.11, 22.68, 22.44, 22.41, 14.11, 9.49. FT-IR (KBr) *v* (cm^−1^): 3310 (N-H), 1630 (C=O), 1110 (O-Si-O). MALDI-TOF MS: calculated for NaC_74_H_147_NO_16_Si_8_ is 1552.88 Da, found: 1552.97.

*8-(2-((3-(3,5,7,9,11,13,15-heptaisobutyl-2,4,6,8,10,12,14,16,17,18,19,20-Dodecaoxa-1,3,5,7,9,11,13,15-octasilapentacyclo[9.5.1.1^3,9^.1^5,15^.1^7,13^]icosan-1-yl)propyl)amino)-2-oxoacetoxy)octyl 3,4,5-tris(dodecyloxy)benzoate (BPOSS-GAD-2)*. White powder. Yield: 46%. ^1^H-NMR (CDCl_3_, ppm): δ 7.25 (s, 2H), 4.28 (m, 4H), 4.01 (t, *J* = 6.4 Hz, 6H), 3.35 (dt, *J*_1_ = 6.8 Hz, *J*_2_ = 6.7 Hz, 2H), 1.89–1.79 (m, 15H), 1.66–1.61 (m, 4H), 1.37–1.27 (br, 62H), 0.97 (d, *J* = 6.6 Hz, 42H), 0.88 (t, *J* = 6.2 Hz, 9H), 0.60 (br, 14H). ^13^C-NMR (CDCl_3_, ppm): δ 166.50, 160.98, 156.38, 152.76, 142.25, 124.98, 107.92, 73.47, 69.12, 67.15, 65.02, 42.09, 31.91, 31.41, 30.93, 30.30, 30.15, 29.68, 29.64, 29.62, 29.56, 29.39, 29.35, 29.28, 29.09, 28.71, 28.29, 26.08, 26.04, 25.88, 25.67, 25.64, 23.86, 23.82, 22.68, 22.43, 22.37, 14.11, 9.37. FT-IR (KBr) *v* (cm^−1^): 3320 (N-H), 1760, 1720, 1680 (C=O), 1100 (O-Si-O). MALDI-TOF MS: calculated for NaC_84_H_163_NO_20_Si_8_ is 1752.98 Da, found: 1752.96.

*4-((8-((3-(3,5,7,9,11,13,15-Heptaisobutyl-2,4,6,8,10,12,14,16,17,18,19,20-dodecaoxa-1,3,5,7,9,11,13,15-octasilapentacyclo[9.5.1.1^3,9^.1^5,15^.1^7,13^]icosan-1-yl)propyl)amino)-8-oxooctanoyl)oxy)phenyl 3,4,5-tris(dodecyloxy)benzoate (BPOSS-GAD-3)*. White powder. Yield: 51%. ^1^H-NMR (CDCl_3_, ppm): δ 7.40 (s, 2H), 7.21(d, *J* = 8.9 Hz, 2H), 7.14 (d, *J* = 8.9 Hz, 2H), 4.05 (dt, *J*_1_ = 6.4 Hz, *J*_2_ = 6.4 Hz, 6H), 3.26 (dt, *J*_1_ = 6.5 Hz, *J*_2_ = 6.5 Hz, 6H), 2.57 (t, *J* = 7.4 Hz, 2H), 2.18 (t, *J* = 7.4 Hz, 2H), 1.89–1.78 (m, 14H), 1.60 (m, 2H), 1.27 (br, 62H), 0.97 (d, *J* = 6.6 Hz, 42H), 0.88 (t, *J* = 6.2 Hz, 9H), 0.62(dd, *J*_1_ = 2.1 Hz, *J*_2_ = 2.1 Hz, 14H). ^13^C-NMR (CDCl_3_, 100 MHz, ppm): δ 172.68, 172.04, 152.92, 142.94, 123.66, 122.59, 122.44, 108.45, 73.56, 69.20, 41.68, 36.74, 34.21, 31.90, 30.31, 29.71, 29.68, 29.64, 29.61, 29.55, 29.37, 29.35, 29.26, 28.94, 28.80, 26.06, 26.03, 25.60, 25.56, 24.67, 23.87, 23.82, 23.04, 22.67, 22.43, 22.41. FT-IR (KBr) *v* (cm^−1^): 3440 (N-H), 1760, 1740, 1650 (C=O), 1110 (O-Si-O). MALDI-TOF MS: calculated for NaC_88_H_163_NO_20_Si_8_ is 1800.98 Da, found: 1800.88.

## 4. Conclusions

In summary, a series of precisely defined shape amphiphiles have been synthesized by covalently linking together POSS and dendrimers with different linker groups. All samples were carefully characterized using NMR, FT-IR, and MALD-TOF MS spectrometry. Rational molecular design can help tune the formation of hierarchical layer structures by adjusting the linkers between two segments of distinct molecular shape. In one of the shape amphiphiles, BPOSS-GAD-3, layered lamellar superlattice was observed with a *d*-spacing around 5.4 nm. We believe that in addition to symmetry breaking and shape incommensurateness, a rigid benzene ring with π-π stacking is critical in stabilizing such structures. The study here will provide insights and comprehensive understanding into the self-assembly and phase separation behavior of shape amphiphiles in general.

## Figures and Tables

**Table 1:** Characterization of POSS-Dendron shape amphiphiles.

Sample	*q* (nm^−1^) ^a^	*d*-Spacing (Å) ^b^	*T_m_* (°C)	Morphology
BPOSS-GAD-1	1.471	42.70	143	crystal
BPOSS-GAD-2	1.203	52.20	74	crystal
BPOSS-GAD-3	1.154	54.42	103	crystal with LAM superlattice

^a^ data read from SAXS. ^b^ data calculated using Bragg’s equation according to *q*.

## Supplementary Materials

Supporting Information is available online.

## References

[1] Hsu C.H., Dong X.H., Lin Z., Ni B., Lu P., Jiang Z., Tian D., Shi A.C., Thomas E.L., Cheng S.Z. (2016). Tunable Affinity and Molecular Architecture Lead to Diverse Self-Assembled Supramolecular Structures in Thin Films. ACS Nano.

[2] Date R.W., Bruce D.W. (2003). Shape Amphiphiles: Mixing Rods and Disks in Liquid Crystals. J. Am. Chem. Soc..

[3] Glotzer S.C., Horsch M.A., Iacovella C.R., Zhang Z., Chan E.R., Zhang X. (2005). Self-assembly of anisotropic tethered nanoparticle shape amphiphiles. Curr. Opin. Colloid Interface Sci..

[4] Feldmann C., Jüstel T., Ronda C.R., Schmidt P.J. (2003). Inorganic luminescent materials: 100 years of research and application. Adv. Funct. Mater..

[5] Zhong Y., Trinh M.T., Chen R., Wang W., Khlyabich P.P., Kumar B., Xu Q., Nam C.-Y., Sfeir M.Y., Black C. (2014). Efficient Organic Solar Cells with Helical Perylene Diimide Electron Acceptors. J. Am. Chem. Soc..

[6] Mulder W.J., Strijkers G.J., van Tilborg G.A., Cormode D.P., Fayad Z.A., Nicolay K. (2009). Nanoparticulate assemblies of amphiphiles and diagnostically active materials for multimodality imaging. Acc. Chem. Res..

[7] Damasceno P.F., Engel M., Glotzer S.C. (2012). Predictive self-assembly of polyhedra into complex structures. Science.

[8] Zhang Z., Horsch M.A., Lamm M.H., Glotzer S.C. (2003). Tethered Nano Building Blocks: Toward a Conceptual Framework for Nanoparticle Self-Assembly. Nano Lett..

[9] Hudson S.D., Jung H.-T., Percec V., Cho W.-D., Johansson G., Ungar G., Balagurusamy V.S.K. (1997). Direct Visualization of Individual Cylindrical and Spherical Supramolecular Dendrimers. Science.

[10] Zeng X., Ungar G., Liu Y., Percec V., Dulcey A.E., Hobbs J.K. (2004). Supramolecular dendritic liquid quasicrystals. Nature.

[11] Ungar G., Liu Y., Zeng X., Percec V., Cho W.D. (2003). Giant supramolecular liquid crystal lattice. Science.

[12] Zhang W.-B., Yu X., Wang C.-L., Sun H.-J., Hsieh I.F., Li Y., Dong X.-H., Yue K., Van Horn R., Cheng S.Z.D. (2014). Molecular Nanoparticles Are Unique Elements for Macromolecular Science: From “Nanoatoms” to Giant Molecules. Macromolecules.

[13] Cordes D.B., Lickiss P.D., Rataboul F. (2010). Recent developments in the chemistry of cubic polyhedral oligosilsesquioxanes. Chem. Rev..

[14] Sun H.-J., Tu Y., Wang C.-L., Van Horn R.M., Tsai C.-C., Graham M.J., Sun B., Lotz B., Zhang W.-B., Cheng S.Z.D. (2011). Hierarchical structure and polymorphism of a sphere-cubic shape amphiphile based on a polyhedral oligomeric silsesquioxane-[60]fullerene conjugate. J. Mater. Chem..

[15] Luo S., Zhang E., Su Y., Cheng T., Shi C. (2011). A review of NIR dyes in cancer targeting and imaging. Biomaterials.

[16] Wang C.L., Zhang W.B., Yu X., Yue K., Sun H.J., Hsu C.H., Hsu C.S., Joseph J., Modarelli D.A., Cheng S.Z. (2013). Facile synthesis and photophysical properties of sphere-square shape amphiphiles based on porphyrin-[60] fullerene conjugates. Chem. Asian J..

[17] Würthner F. (2004). Perylene bisimide dyes as versatile building blocks for functional supramolecular architectures. Chem. Commun..

[18] Wang C.L., Zhang W.B., Van Horn R.M., Tu Y., Gong X., Cheng S.Z., Sun Y., Tong M., Seo J., Hsu B.B. (2011). A porphyrin–fullerene dyad with a supramolecular “double-cable” structure as a novel electron acceptor for bulk heterojunction polymer solar cells. Adv. Mater..

[19] Huang C., Barlow S., Marder S.R. (2011). Perylene-3,4,9,10-tetracarboxylic acid diimides: Synthesis, physical properties, and use in organic electronics. J. Org. Chem..

[20] Liang W.-W., Huang C.-F., Wu K.-Y., Wu S.-L., Chang S.-T., Cheng Y.-J., Wang C.-L. (2016). Flat-on ambipolar triphenylamine/C60 nano-stacks formed from the self-organization of a pyramid-sphere-shaped amphiphile. Chem. Sci..

[21] Kaiser T.E., Wang H., Stepanenko V., Würthner F. (2007). Supramolecular Construction of Fluorescent J-Aggregates Based on Hydrogen-Bonded Perylene Dyes. Angew. Chem. Int. Ed..

[22] Ren X., Sun B., Tsai C.-C., Tu Y., Leng S., Li K., Kang Z., Horn R.M.V., Li X., Zhu M. (2010). Synthesis, Self-assembly, and Crystal Structure of a Shape-Persistent Polyhedral-Oligosilsesquioxane-Nanoparticle-Tethered Perylene Diimide. J. Phys. Chem. B.

[23] Yu X., Yue K., Hsieh I.F., Li Y., Dong X.H., Liu C., Xin Y., Wang H.F., Shi A.C., Newkome G.R. (2013). Giant surfactants provide a versatile platform for sub-10-nm nanostructure engineering. Proc. Natl. Acad. Sci. USA.

[24] Huang M., Hsu C.H., Wang J., Mei S., Dong X., Li Y., Li M., Liu H., Zhang W., Aida T. (2015). Self-assembly. Selective assemblies of giant tetrahedra via precisely controlled positional interactions. Science.

[25] Cui L., Collet J.P., Xu G., Zhu L. (2006). Supramolecular Self-Assembly in a Disk-Cube Dyad Molecule Based on Triphenylene and Polyhedral Oligomeric Silsesquioxane (POSS). Chem. Mater..

[26] Liu H., Hsu C.H., Lin Z., Shan W., Wang J., Jiang J., Huang M., Lotz B., Yu X., Zhang W.B. (2014). Two-dimensional nanocrystals of molecular Janus particles. J. Am. Chem. Soc..

[27] Liu H., Luo J., Shan W., Guo D., Wang J., Hsu C.H., Huang M., Zhang W., Lotz B., Zhang W.B. (2016). Manipulation of Self-Assembled Nanostructure Dimensions in Molecular Janus Particles. ACS Nano.

[28] Custers J.P.A., Hersmis M.C., Meuldijk J., Vekemans J.A.J.M., Hulshof L.A. (2002). 3,4,5-Tri-Dodecyloxybenzoic Acid: Combining Reaction Engineering and Chemistry in the Development of an Attractive Tool to Assist Scaling Up Solid–Liquid Reactions. Org. Process Res. Dev..

[29] Percec V., Aqad E., Peterca M., Rudick J.G., Lemon L., Ronda J.C., De B.B., Heiney P.A., Meijer E.W. (2006). Steric communication of chiral information observed in dendronized polyacetylenes. J. Am. Chem. Soc..

[30] Asahi Kasei Chemicals Corporation (2010). Process for Production of Powder of Cage Silsesquioxane Compound. Patent.

[31] Li Y., Zhang W.B., Hsieh I.F., Zhang G., Cao Y., Li X., Wesdemiotis C., Lotz B., Xiong H., Cheng S.Z. (2011). Breaking symmetry toward nonspherical Janus particles based on polyhedral oligomeric silsesquioxanes: Molecular design, “click” synthesis, and hierarchical structure. J. Am. Chem. Soc..

