# Supplementary material for: Synthesis and Self-Assembly of Shape Amphiphiles Based on POSS-Dendron Conjugates
Source: Molecules. 2017 Apr 21;22(4):622. doi: 10.3390/molecules22040622 (PMC6154716; doi:10.3390/molecules22040622)
Supplement: Supplementary file 1 [file molecules-22-00622-s001.pdf]

Supplementary Materials

Synthesis and Self-assembly of Shape Amphiphiles Based on POSS-Dendron Conjugates

Yu Shao, Minyuan Ding, Fangjia Zhao, Hui Dai, Xia-Ran Miao, Shuguang Yang, Hui Li

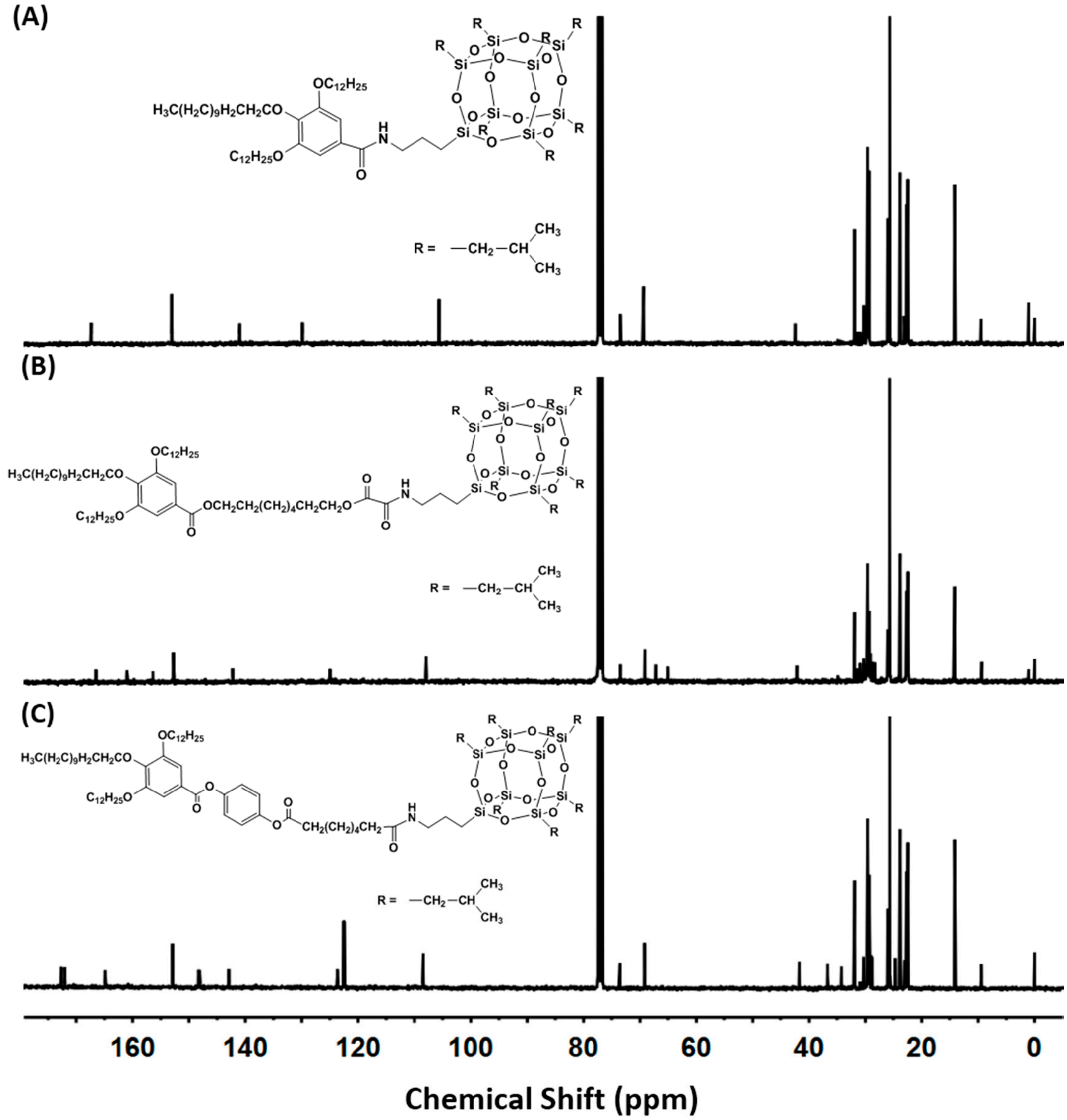

Figure S1. ^{13}C-NMR spectrum of BPOSS-GAD-1 (A), BPOSS-GAD-2 (B) and BPOSS-GAD-3 (C).

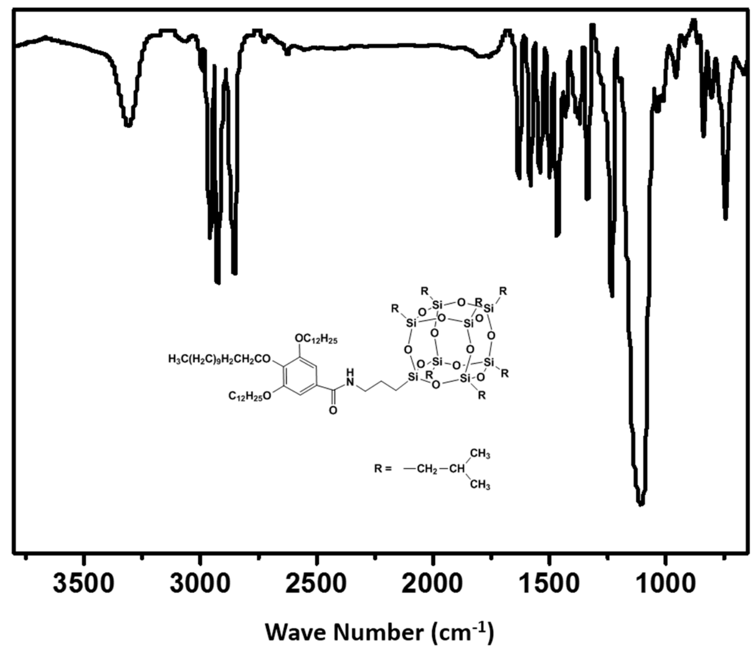

Figure S2. FT-IR spectrum of BPOSS-GAD-1

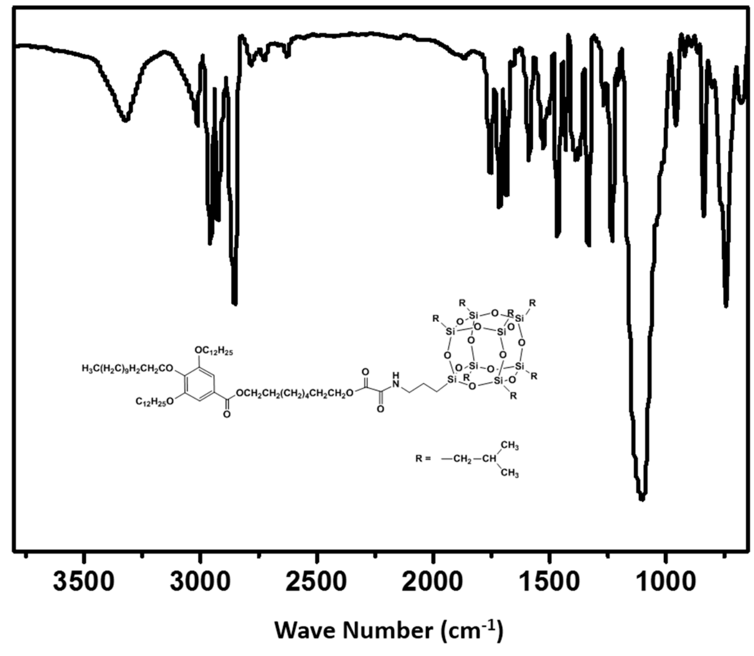

Figure S3. FT-IR spectrum of BPOSS-GAD-2

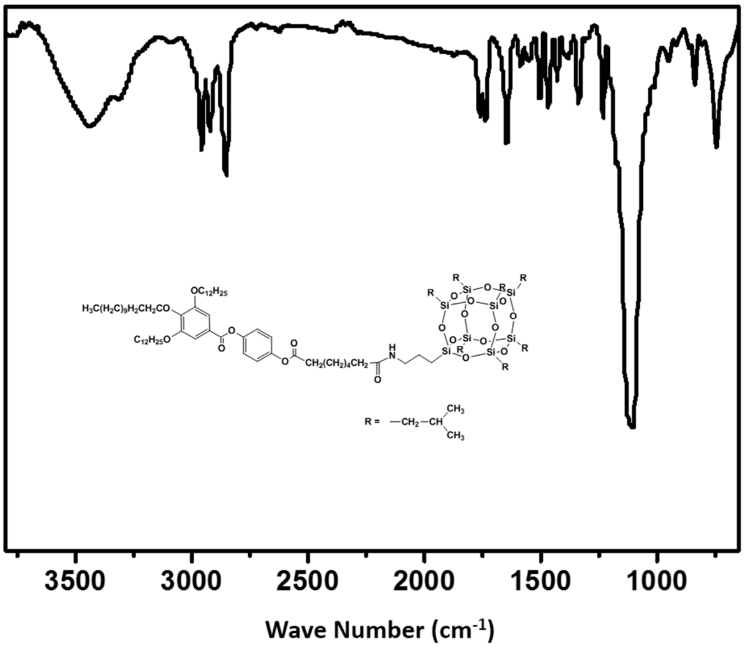

Figure S4. FT-IR spectrum of BPOSS-GAD-3

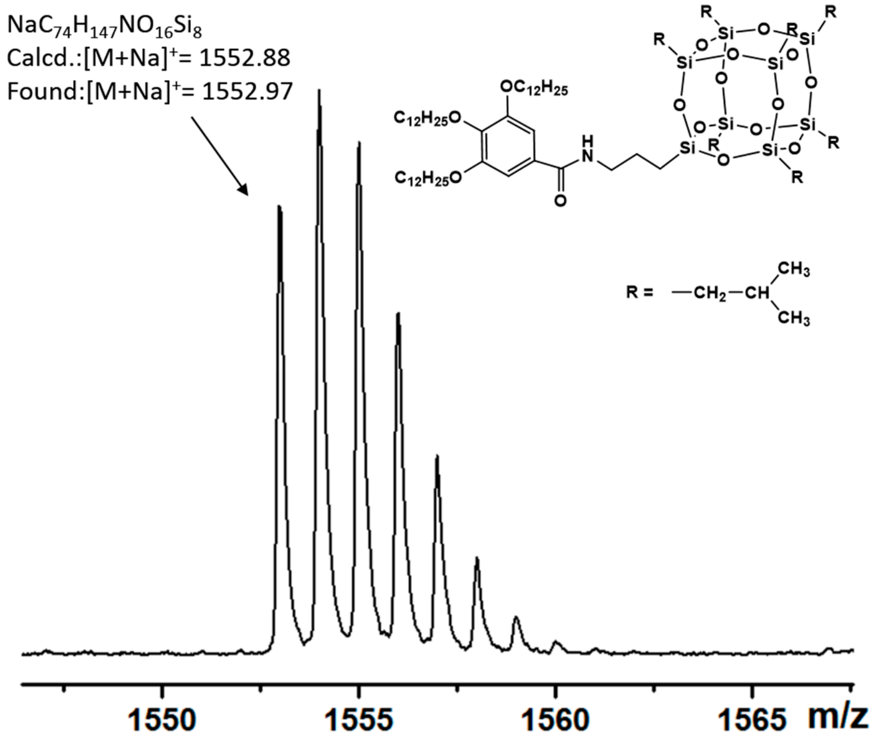

Figure S5. MALDI-TOF MS spectrum of BPOSS-GAD-1

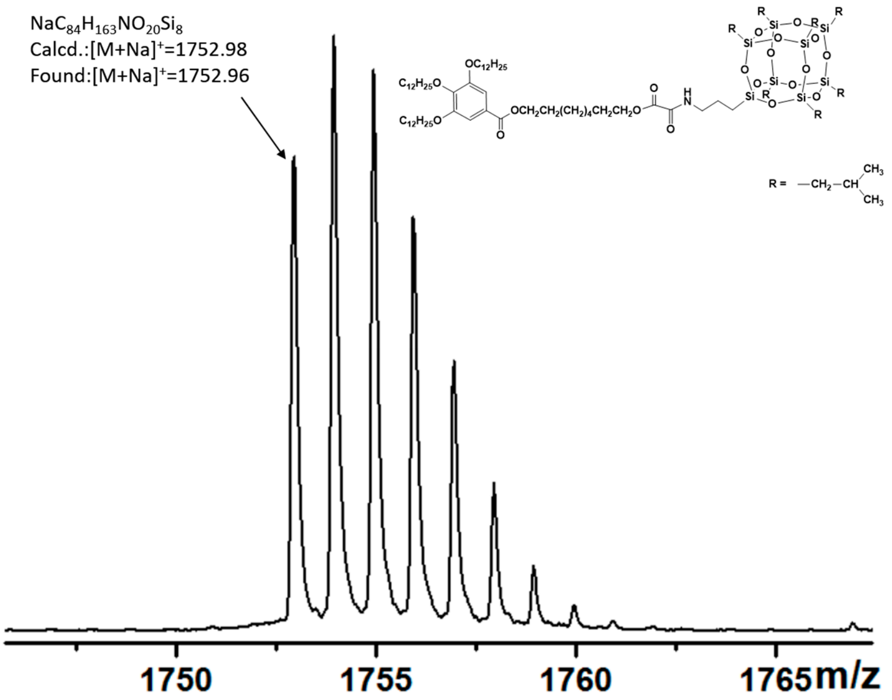

Figure S6. MALDI-TOF MS spectrum of BPOSS-GAD-2

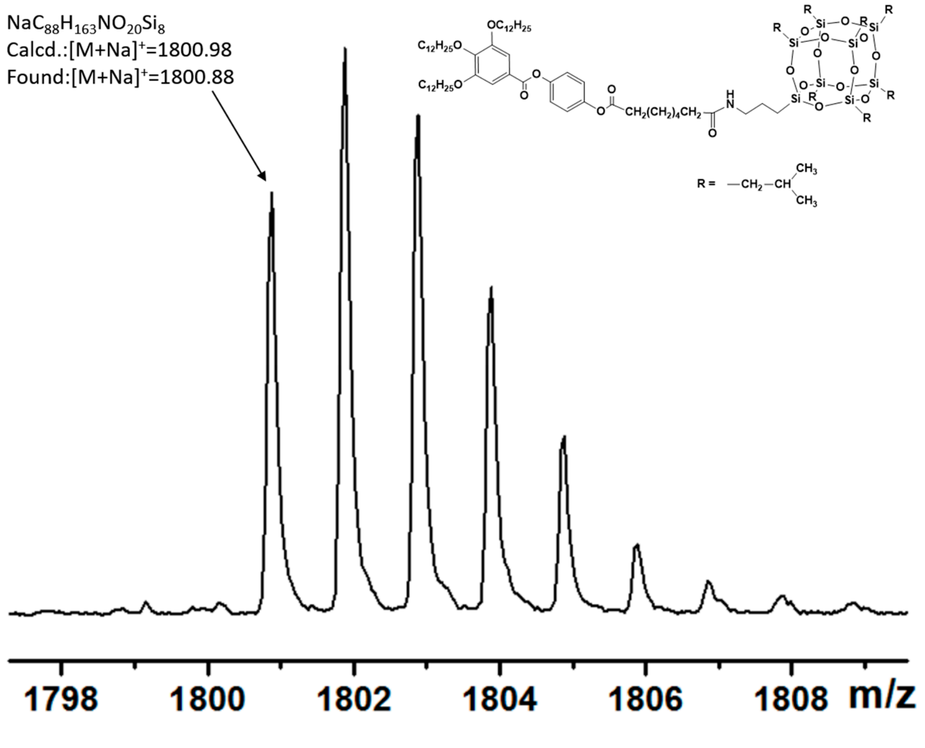

Figure S7. MALDI-TOF MS spectrum of BPOSS-GAD-3

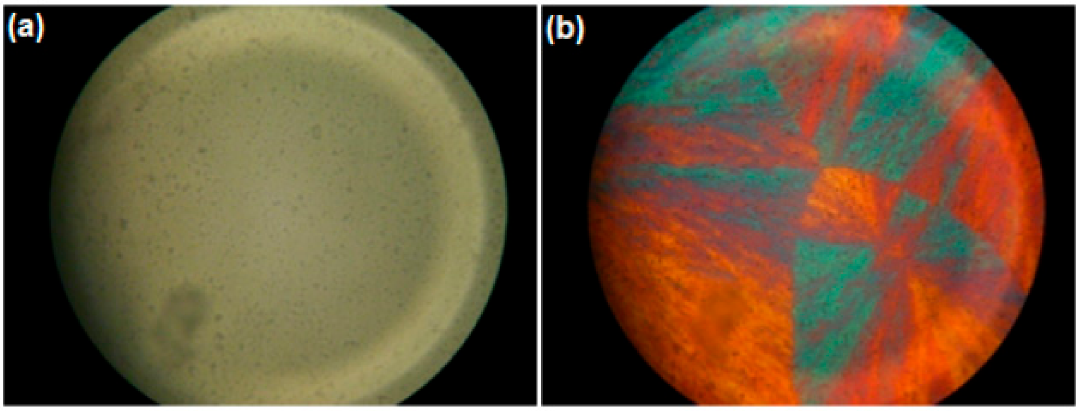

Figure S8. PLM pictures of BPOSS-GAD-1, (a) 180 ºC and (b) 30 ºC.

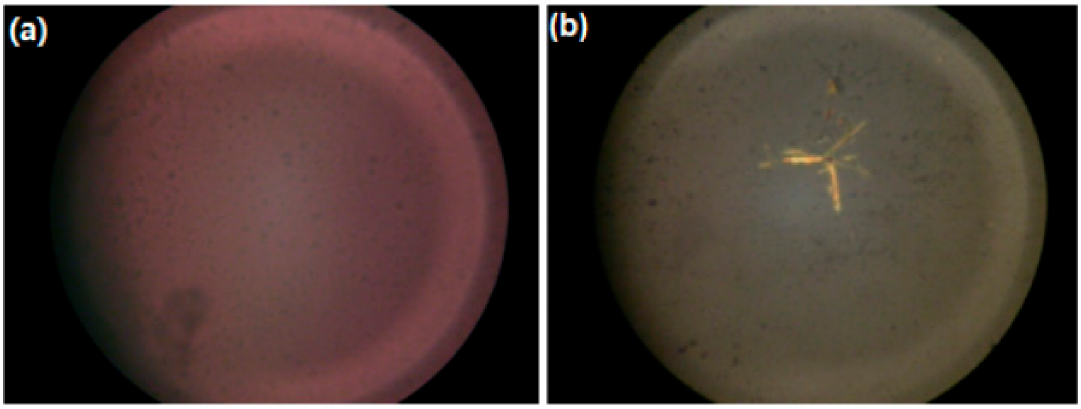

Figure S9. PLM pictures of BPOSS-GAD-2, (a) 180 ºC and (b) 30 ºC.

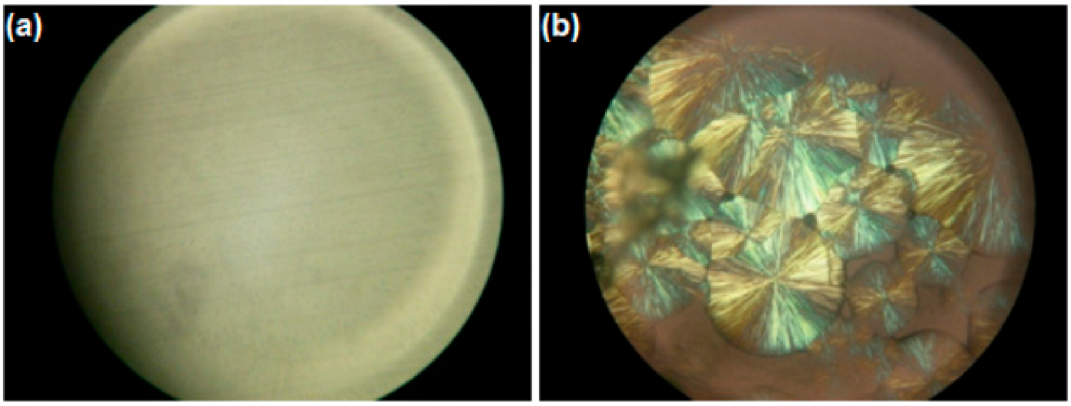

Figure S10. PLM pictures of BPOSS-GAD-3, (a) 180 ºC and (b) 30 ºC.
